# Continuous and Discontinuous Cigarette Smoke Exposure Differentially Affects Protective Th1 Immunity against Pulmonary Tuberculosis

**DOI:** 10.1371/journal.pone.0059185

**Published:** 2013-03-19

**Authors:** Christopher R. Shaler, Carly N. Horvath, Sarah McCormick, Mangalakumari Jeyanathan, Amandeep Khera, Anna Zganiacz, Joanna Kasinska, Martin R. Stampfli, Zhou Xing

**Affiliations:** McMaster Immunology Research Centre, and Department of Pathology & Molecular Medicine, McMaster University, Hamilton, Ontario, Canada; Glaxo Smith Kline, Denmark

## Abstract

Pulmonary tuberculosis (TB), caused by *Mycobacterium tuberculosis,* is the leading cause of death due to a bacterial pathogen. Emerging epidemiologic evidence suggests that the leading risk factor associated with TB mortality is cigarette smoke exposure. Despite this, it remains poorly understood what is the effect of cigarette smoke exposure on anti-TB immunity and whether its potential detrimental effect can be reversed by cigarette smoking cessation. In our current study, we have investigated the impact of both continuous and discontinuous cigarette smoke exposure on the development of anti-mycobacterial type 1 immunity in murine models. We find that while continuous cigarette smoke exposure severely impairs type 1 immunity in the lung, a short-term smoking cessation allows rapid restoration of anti-mycobacterial immunity. The ability of continuous cigarette smoke exposure to dampen type 1 protective immunity is attributed locally to its affects on innate immune cells in the lung. Continuous cigarette smoke exposure locally, by not systemically, impairs APC accumulation and their production of TNF, IL-12, and RANTES, blunts the recruitment of CD4+IFN-γ+ T cells to the lung, and weakens the formation of granuloma. On the other hand, smoking cessation was found to help restore type 1 immunity by rapidly improving the functionality of lung APCs, enhancing the recruitment of CD4+IFN-γ+ T cells to the lung, and promoting the formation of granuloma. Our study for the first time demonstrates that continuous, but not discontinuous, cigarette smoke exposure severely impedes the lung expression of anti-TB Th1 immunity via inhibiting innate immune activation and lung T cell recruitment. Our findings thus suggest cigarette smoking cessation to be beneficial to the control of pulmonary TB.

## Introduction

Globally, tuberculosis (TB) represents a leading public heath concern with one third of the world’s population latently infected [1]. Despite the prevalence of individuals infected with *Mycobacterium tuberculosis (M.tb)*, only 5–10% of them go to develop the active disease [1]. While the majority of TB cases are seen in the developing world, developed nations are not immune. Notably, TB is common among Native American, prison and homeless populations, where incidence rates are very similar to those seen in the developing world [2], [3].

The influence of HIV-AIDS on TB has long been acknowledged and is commonly perceived as a leading risk factor in the developing world. Indeed, HIV-AIDS accounts for one quarter of all TB-related deaths [1]. While nutrition, population density, and access to advanced health care are significant factors in the development of active disease, the leading risk factor associated with acquisition, active disease and mortality is the exposure to tobacco smoke [4]. Active smoking and exposure to second hand smoke which is a significant concern especially in children [5], [6], account for approximately 60% of all TB related deaths. Alarmingly, the consumption of tobacco products has skyrocketed in the developing world. Current estimates suggest that 85% of all cigarettes are now being consumed in the developing world, with the highest numbers in regions where TB is rampant [7]. The collision of these two epidemics makes unraveling how cigarette smoke exposure impacts TB immunity a particularly relevant challenge.

Protective immunity to *M.tb* largely relies on the generation of a robust type 1 immune response, requiring the elaborate coordination of the innate and adaptive immune systems. Following exposure, *M.tb* primarily infects the alveolar macrophage (AM), utilizing the cell’s phagocytic machinery to facilitate its uptake. The infected macrophage detects *M.tb* through the engagement of pattern recognition receptors, specifically toll like receptors (TLRs) 2, 4 & 9, triggering the release of various pro-inflammatory cytokines [8]. Notably, the production of TNF and IL-12 is critical to bridging the innate and adaptive immune systems. Acting as an alarm cytokine TNF plays a central role in coordinating the release of chemokines and the recruitment of innate immune cells to the lung. After acquiring antigen in the lung, recruited APCs (antigen presenting cells) migrate to the draining lymph node (dLN) to present antigen to naïve T cells, initiating the cellular immune response. At the time of antigen presentation, the release of IL-12 is essential to correctly polarizing Th1 responses, and an absence of IL-12 is detrimental to generating protective immunity [9]. Similarly, a failure to establish strong chemokine gradients prevents T cells from homing to the lung and is equally detrimental to establishment of protective immunity, as has been seen in RANTES deficient mice [10].

Cigarette smoke’s impact on immunity is complex; cigarette smoke exerts damaging and pro-inflammatory effects, while suppressing components of both innate and adaptive immunity (reviewed in Stampfli and Anderson [11]). While cigarette smoke activates the AM, cigarette smoke attenuates the expression of key inflammatory mediators such as IL-12, TNF and RANTES that play a critical role in anti-TB host defense. Furthermore, evidence suggests that cigarette smoke impairs the generation of type 1 immunity, leaving infected hosts highly susceptible to certain viral and bacterial pathogens [12], [13].

Recently, two groups have begun to address the impact of cigarette smoke exposure on the development of type 1 immunity in the context of *M.tb* or mycobacterial infection in experimental models [14], [15]. While these studies have demonstrated a link between cigarette smoke exposure and impaired type 1 immunity in the lung, they have only assessed the impact of prior (discontinued) cigarette smoke exposure on anti-TB immunity. To date no study has evaluated the effect of continuous cigarette smoke exposure, relative to discontinued cigarette smoke exposure, on host defense against pulmonary mycobacterial infection, leaving a critical knowledge gap.

In the current study, we addressed this significant knowledge gap and investigated the impact of both continuous and discontinuous cigarette smoke exposure on the generation of protective immunity following mycobacterial challenge. We have evaluated the effect of cigarette smoke exposure on immune responses generated both locally in the lung and distally in the draining lymph nodes and spleen. Our study has revealed a profound negative effect of continuous, but not prior (discontinuous), cigarette smoke exposure on host defense mechanisms in the lung with a much less effect in the systemic tissue compartments.

## Materials and Methods

### Ethic Statement

All animal experiments including animal care and procedures were conducted in accordance with the guidelines from the Canadian Council on Animal Care. This study was approved by the Animal Research Ethics Board of McMaster University with an animal utilization protocol number 10-04-23.

### Mice

Female C57BL/6 mice (6–8 wk old) were purchased from Charles River Laboratories (Charles River, St Constant, Quebec, Canada) and housed in a specific pathogen-free, level B facility for the duration of cigarette smoke exposure. Following cigarette smoke exposure mice were either infected with *Mycobacterium bovis* Bacille Calmette Guerien (BCG) and housed under level II bio-hazardous conditions, or *M.tb* H_37_Rv and housed at level III bio-hazardous conditions, all mice were maintained in specific pathogen-free environments regardless of containment level. All animals were maintained on a constant light: dark 12∶12 cycle and given free access to food and water. For all experiments, mice were euthanized by exsanguination of the abdominal artery under anesthesia.

### Cigarette Smoke Exposure

Using a whole body exposure system (SIU-48, Promech Lab AB, Vintrie, Sweden), mice were exposed to cigarette smoke as previously described [16], [17], [18]. In brief mice were exposed twice daily for 50 mins, 5 days a week to 12 2R4F reference cigarettes (Tobacco and Health Research Institute, University of Kentucky, Lexington, KY, USA) with filters removed. Mice were exposed to cigarette smoke (or room air) for 6 wks prior to mycobacterial infection. At the time of infection, one group of exposed mice stopped cigarette smoke exposure (cessation), while the other group continued for the duration of the experimental infection, leading to an exposure of 6 or 10 wks, respectively. This protocol of cigarette smoke exposure has been validated and shown to achieve blood carboxyhaemoglobin and cotinine levels that are comparable to those found in regular human smokers [19].

### Mycobacterial Preparation and Infectious Dose

*Mycobacterium bovis* BCG (Connaught strain) was prepared as previously described [9], [20]. Briefly, BCG was grown in Middlebrook 7H9 broth (Difco) supplemented with Middlebrook OADC enrichment (Invitrogen), 20% glycerol, and 0.05% Tween 80 for 10 to 15 days, and samples were then divided into aliquots and stored at –70°C. Before each use, a BCG aliquot was washed twice with phosphate-buffered saline (PBS) containing 0.05% Tween 80 and resuspended in PBS. It was then passed through a 27-gauge needle 10 times to disperse clumps and diluted with PBS to the desired concentration. Mice were infected intratracheally with a dose of 0.5×10^6^ cfu/mouse for elicitation of strong Th1 immune responses and granuloma formation.

*Mycobacterium tuberculosis* H_37_Rv was prepared and processed as described above for BCG. Mice were infected with *M.tb* intranasally by using a dose of 10000 CFU/mouse as previously described by us (depositing 1000±150 CFU into the lung). A higher inoculum of BCG was used to compensate for its attenuated virulence nature, allowing generation of Th1 immune responses highly similar to those seen following *M.tb* challenge. These doses have previously been shown to elicit significant Th1 immunity and the formation of robust granulomas [21], [22].

### Cell Culture and Cytokine Measurement

Total airway luminal, lung interstitial, spleen or mononuclear cells (MLN) (0.25×10^6^/well) were seeded into a 96-well flat bottom plate and cultured at 37°C and 5% CO_2_ with or without mycobacterial antigen stimulation for 48 hrs. The antigens used for stimulation were *M. tuberculosis* culture filtrate proteins (*M.tb*-CF) (2 µg/well). Cells were cultured in a total volume of 250 µl of cRPMI. Culture supernatants were collected at 48 hours and stored at −20°C until cytokine/chemokine measurement. TNF-α, IFN-γ IL-12p40 and IL-10 concentrations were measured by using duoset ELISA kits (R&D systems).

### Nitric Oxide Production Measurement

The release of nitric oxide (NO) by lung derived cells was determined by measuring the end product of NO, nitrite, as previously described [23]. Briefly, diluted supernatants were added at a 1∶1 ratio with Griess reagent buffer (Sigma-Aldrich). The absorbance was measured at 540 nm by a spectrophotometer. The final concentration of nitrite was calculated by referring to a standard curve prepared from 0 to 100 µM of sodium nitrite concentrations.

### Cell Surface Immunostaining and Intracellular Cytokine Staining (ICCS)

All monoclonal antibodies (mAbs) used were purchased from BD Pharmingen. Immunostaining and FACS were carried out as previously described [9], [24], [25]. Briefly, cells were blocked for non-specific binding of their Fc receptors with anti-CD16/CD32 antibodies for 15 min and then stained for 30 min on ice with the appropriate combinations of fluorochrome-conjugated mAbs. Fluorochrome-conjugated mAbs to CD11b, CD11c, CD3, CD4, and CD8 were used. Appropriate controls were used for each antibody. For intracellular cytokine staining (ICCS), single cell suspensions from airway lumen, lung, spleen and MLN were cultured and stained as previously described [26]. Briefly, cells were cultured for 24 hours with or without mycobacterial antigens (*M.tb*-Culture Filtrate-CF and crude BCG), Golgi Plug (5 µg/ml brefeldin A BD Bioscience, Burlington, Ontario, Canada) was added 18 hours after stimulation. After culture, cells were washed and blocked with CD16/CD32 for 15 min on ice and stained with cell surface Abs. In some experiments, cells were then washed, permeabilized and stained with IFN-γ and IL-4, or TNF and IL-12, Abs according to the manufacturer’s instructions included in the ICCS kit (BD Pharmingen). Stained cells were run on the LSRII (BD Biosciences) flow cytometer using FACSDiva software and data was analyzed with Flowjo software (Tree Star, Ashland, OR). Depending on the number of cells available, 100,000 to 250,000 events per sample were analyzed.

### Bacterial Enumeration and Lung Histology

The bacterial load in the lung and spleen were enumerated as previously described. Briefly, half lungs and whole spleens were sterilely collected at the time of sacrifice and homogenized in PBS. Lung and spleen homogenates were subjected to serial dilution and plated on Middlebrook 7H10 agar plates, supplemented with Middlebrook OADC enrichment (Invitrogen). Bacterial plates were incubated at 37°C for 15–17 days until colonies were visible, at which time colonies were enumerated and the bacterial burden at time of sacrifice calculated.

For the evaluation of histological changes the left lungs of infected mice were isolated sectioned and stained with haematoxylin and eosin. Stained sections were mounted to slides and histological evaluation was performed by conventional light microscopy at various magnification (5×, 10×, 20×), looking for structurally and morphological changes associated with cigarette smoke exposure and/or mycobacterial infection. Histological sections were blindly scored for lung inflammation, cellular infiltration and granuloma formation using 5× magnification H&E stained lung sections. Three sections were evaluated per mouse lung, with 4–5 mice evaluated per group.

### Statistical Analysis

Statistical analysis was performed using either one-way anova, or unpaired two-tailed student’s *t* test using the statistical analysis component of GraphPad Prism software. Values of p<0.05 were considered statistically significant.

## Results

### Continuous, but not Discontinued, Cigarette Smoke Exposure Significantly Impairs Bacterial Control Following Pulmonary Mycobacterial Infection

In support of epidemiological data suggests that cigarette smoke significantly impacts the host’s ability to control *M.tb* [5], [6], [14], [15], experimental models have shown that mice exposed to cigarette smoke prior to *M.tb* infection reduces the host’s ability to control bacterial growth and prevent dissemination [14], [15]. However, to date no model has compared the impact of both prior and continuous cigarette smoke exposure on the development of anti-mycobacterial immunity and bacterial control. To address this question, we first established and characterized a 6-wk continuous cigarette smoke exposure model (Figure S1A). Cigarette smoke exposure significantly increased alveolar macrophages, neutrophils and lymphocytes in the airway lumen (Figure S1B/S1C). Consistent with increased inflammatory cells in the airway lumen was increased inflammatory cellularity in the bronchial epithelium and alveolar septa (Figure S1D–G). By using FACS, compared to sham, room air-exposed animals, the total lung mononuclear cells from cigarette smoke-exposed animals, contained a marked increased number of activated macrophages (CD11b+CD11c+) and neutrophils (CD11b+GR1+) and a F4/80+ cell population consistent with the phenotype of newly recruited macrophages (Figure S2A/S2B).

**Figure 1:**
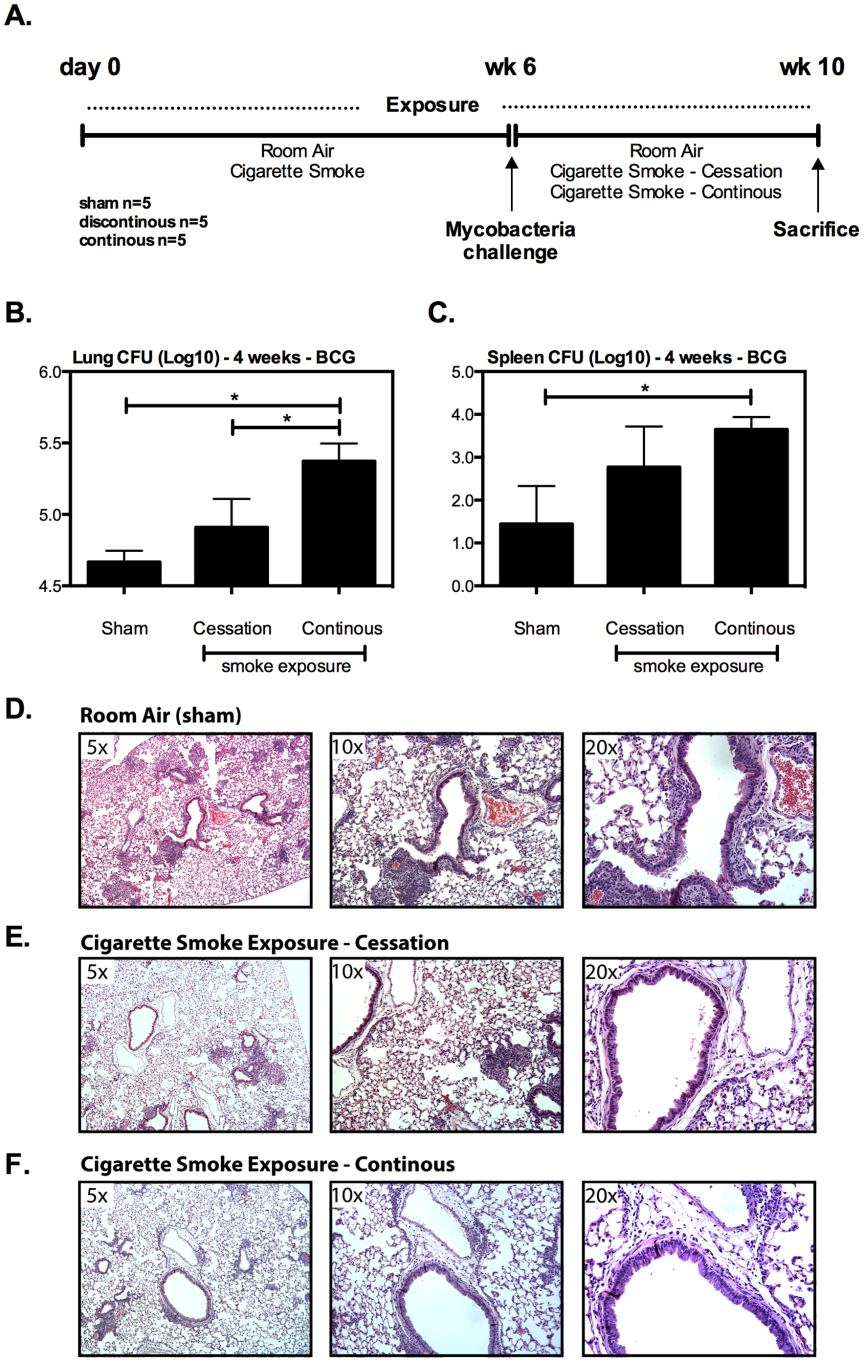
Continuous cigarette smoke exposure alters lung pathology and decreases bacterial control following pulmonary mycobacterial infection. Following 6 wks of cigarette smoke (or room air) exposure, mice were subjected to Bacillus Calmette–Guérin - *M. bovis* challenge (A). At the time of challenge one group of previously cs exposed mice was discontinued from cigarette smoke exposure to determine the impact of cessation of mycobacterial immunity, while another continued exposure for the duration of infection. At 4 wks post-infection, the bacterial burden following the various exposure protocols was determined by colony formation assay in the lung and spleen of mycobacterial infected mice (B&C), and the histological impact on lung pathology by H&E staining of lung sections isolated from infected mice (D–F). CFU numbers represents the mean and standard error of 5 mice per exposure protocol. Selected histological sections are representative of their exposure protocol. *p≤0.05.

**Figure 2:**
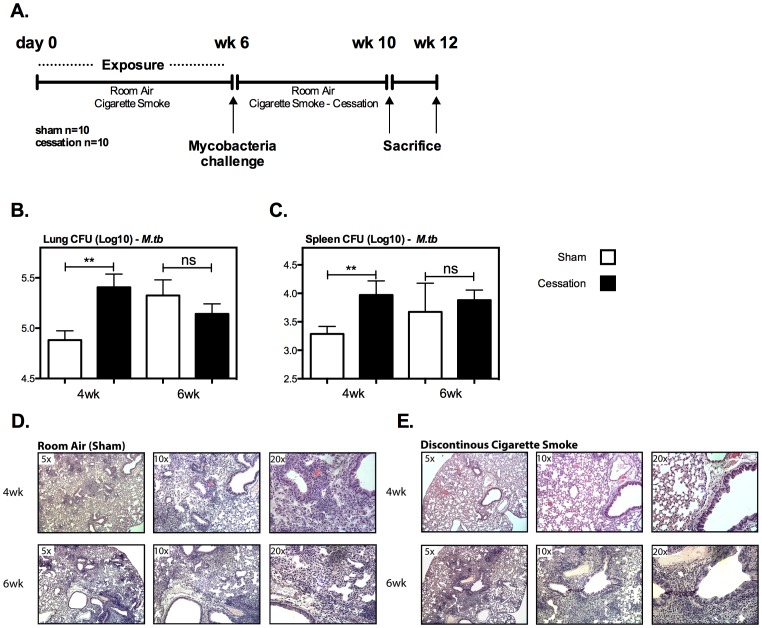
Prolonged cigarette smoke cessation enhances cellular infiltration, granuloma formation and bacterial control following mycobacterial challenge. Mice where either exposed to cigarette smoke or room air for a period of 6 wks, at which time both groups were subjected to challenge with *M.tb* H_37_Rv (A). The bacterial burden was determined in the lung (B), and spleen (C) by colony formation assay of organ homogenates from infected mice at 4 and 6 wk post infection. The histological impact on lung pathology was determined by H&E staining of lung sections isolated 4 and 6 wks post infection with *M.tb* (D&E). CFU numbers represent the mean and standard error of 5 mice per exposure protocol. The 4 wk challenge data is representative of two independent experiments. Selected histological sections are representative of their exposure protocol. *p≤0.05; **p≤0.01.

To investigate the impact of continuous and discontinuous cigarette smoke exposure on anti-mycobacterial host defense, mice are exposed to cigarette smoke or room air for a period of 6 wks and subsequently infected with mycobacterial BCG (Figure 1A), at which time cigarette smoke was discontinued (cessation model) for one group, while being continued until sacrifice in another (continuous exposure model). Continuous cigarette smoke exposure most significantly impaired bacterial control both locally in lung and systemically in the spleen (Figure 1B and 1C). However, by comparison cigarette smoke cessation (prior cigarette smoke exposure) partially restored mycobacterial control in both the lung and spleen (Figure 1B and 1C). Noting that cigarette smoke cessation for 4 wks significantly improved bacterial control, we sought to evaluate whether prolonged cessation would allow for prior cigarette smoke exposed mice to regain further improved bacterial control similar to that seen in room air exposed mice (Figure 2A). Indeed, compared to a 4-wk smoke cessation interval, at a 6-wk interval post-*M.tb* infection the bacterial control of prior cigarette smoke exposed mice was comparable to that of room air controls (Figure 2B/2C). The profound ability of cigarette smoking cessation to improve bacterial control indicates that continuous cigarette smoke exposure is required to maintain a robust suppressive effect on anti-mycobacterial immunity.

BCG immunization is implemented in most of the developing countries and it enhances anti-TB immunity in the lung of experimental animals [21]. As we have seen the improved protective immunity following cigarette smoking cessation (Figures 1 and 2), we examined whether this could also be the case in prior BCG-immunized animals. Using the model outlined in Figure S3A, BCG-immunized mice that had previously been exposed to cigarette smoke, demonstrated a level of enhanced protection in the lung and spleen from *M.tb* challenge similar to that in *M.tb*-challenged room air (sham) exposed animals (Figure S3B/S3C). However, prior cigarette smoke exposure increased lung pathology in response to *M.tb* infection (Figure S3D–F). These results suggest that like in unimmunized hosts, cigarette smoking cessation helps restore protective immunity in the lung and spleen but at the expense of causing more pronounced lung pathology.

### Continuous Cigarette Smoke Exposure Abrogates the Generation of Granuloma Formation and T Cell Immunity in the Lung during Pulmonary Mycobacterial Infection

To date little is known about how continuous cigarette smoke exposure influences the development of lung pathology during mycobacterial infection. Using the above described exposure models (Figure 1A) we set out to determine the effect of cigarette smoke exposure on the development of lung granuloma and tissue inflammatory responses. At 4 wks following mycobacterial infection compared to vigorous tissue inflammation seen in the lung of room air exposure (Figure 1D), continuous cigarette smoke exposure markedly depressed cellular infiltration and granuloma formation in the lungs of mycobacterial-infected mice (Figure 1F; Table 1). Cigarette smoke cessation was found to increase cellular infiltration compared to continuous cigarette smoke exposure although the extent of infiltration did not reach what was seen in room air exposed mice (Figure 1D/1E; Figure 2D/2E; Tables 1 and 2).

**Table 1:** Assessment of Histopathological Changes in the Lung following BCG challenge.

	Room Air (sham)	Discontinuous	Continuous
**size granuloma**	+++	+++	++
**number of granuloma**	+++	+++	++
**cellular infiltration**	++++	+++	++

Granuloma size, granuloma number, and lung mononuclear cell infiltration were scored.

Results are representative of *n*  = 5 mice/exposure/time point.

+, minimal; ++, slight; +++, moderate; ++++, marked; (+ half point).

**Table 2:** Assessment of Histopathological Changes in the Lung following *M.tb* challenge.

	4 wk		6 wk	
	Sham	Discontinuous	Sham	Discontinuous
**size** **granuloma**	++++	+++	+++++	++++
**number of granuloma**	+++++	+++	+++++	++++
**cellular** **infiltration**	+++++	+++	+++++	+++++

Granuloma size, granuloma number, and lung mononuclear cell infiltration were scored.

Results are representative of *n*  = 5/exposure/time point.

+++, moderate; ++++, marked; +++++, severe. (+ half point).

Having noted that cigarette smoke cessation restored bacterial control by 6 wks post-*M.tb* challenge (Figure 2B/2C), we wondered whether cellular infiltration and granuloma formation was similarly restored. As anticipated, by 6 wks of cigarette smoke cessation the mice showed similar levels of lung cellular infiltration to room air controls, with notable granuloma formation (Figure 2D/2E; Table 2).

**Figure 3:**
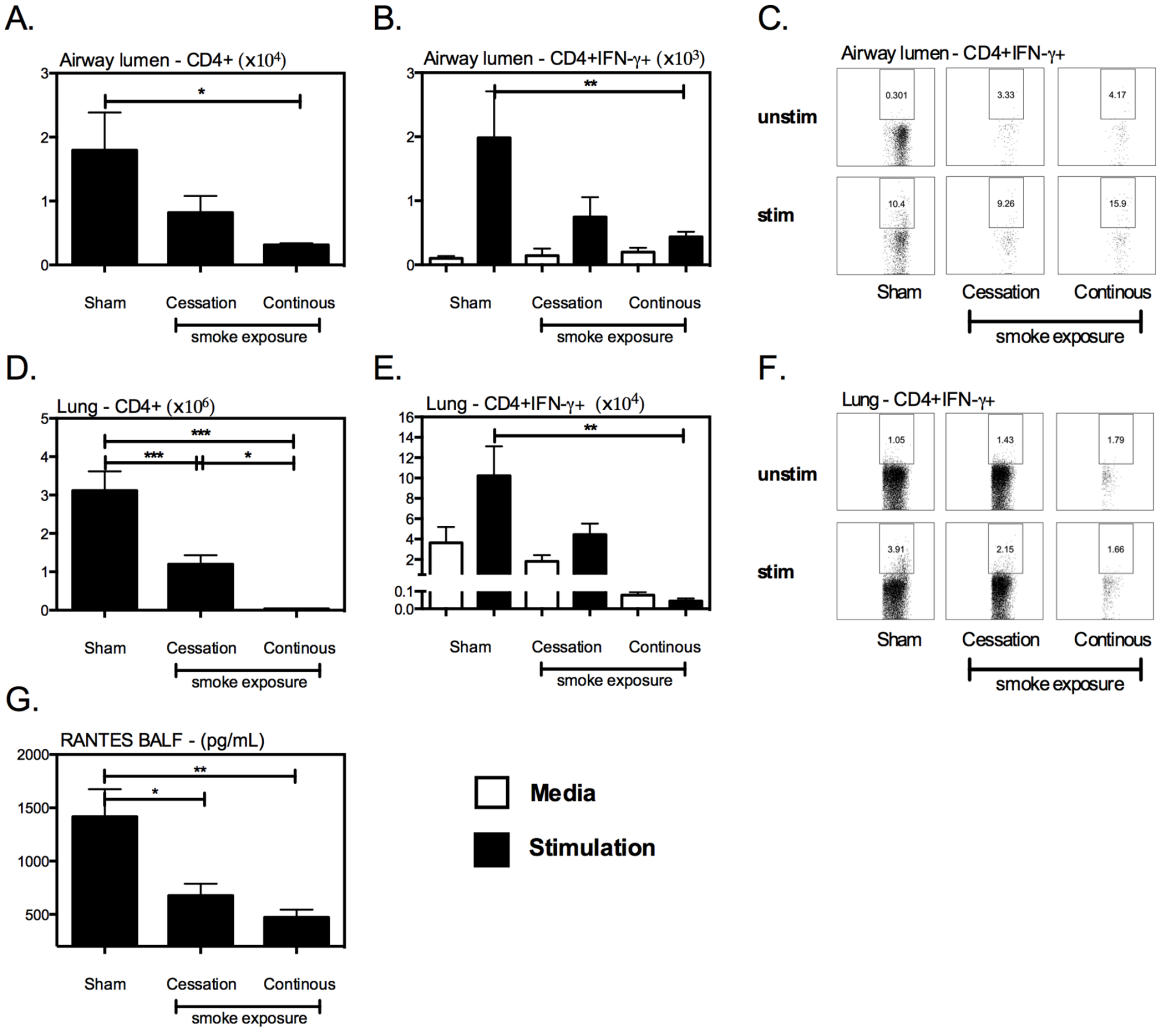
Continuous cigarette smoke exposure impairs the establishment of type 1 immunity in the lung of mycobacteria infected mice. Following the exposure-challenge model described in Figure 2A, we evaluated the impact of cigarette smoke exposure on the establishment of type 1 immune response in the lung of mycobacterial infected mice. The numbers or frequencies of CD4+ and CD4+IFN-γ+ T cells were evaluated in the airway lumen (A/B/C), and the lung interstitium (D/E/F). The levels of RANTES were assessed in bronchoalveolar lavage fluids (BALFs) (G). Values represent the mean and standard error for 5 mice per exposure protocol. The dotplots are representative images from their respective groups. *p≤0.05; **p≤0.01; ***p≤0.001.

Given that continuous cigarette smoke exposure led to significantly impaired granulomatous inflammation in the lung following mycobacterial infection (Figure 1F), we examined whether it impacted the development of T cell immunity. In accordance with their severely impaired lung protection, the mice that were continuously exposed to cigarette smoke showed a pronounced defect in the accumulation of T cells in the lung. Continuous cigarette smoke exposure resulted in profound lymphopenia (lack of total CD4 T cells) in the lung (Figure 3A/3D), with virtually undetectable CD4+ IFN-γ+ T cell responses in both the airway lumen and lung interstitium (Figure 3B/3E/3C/3F). In contrast to continuous cigarette smoke exposure, by 4 wks cigarette smoking cessation had partially restored the recruitment of CD4+ IFN-γ+ T cells into the lung, resulting in an increase in both the frequency (Figure 3C/3F) and total numbers of CD4+IFN-γ+ T cells (Figure 3B/3E). Previously we have documented that cigarette smoke exposure significantly hampers the production of a critical T cell chemokine, RANTES (CCL5), by alveolar macrophages exposed to cigarette smoke [18]. Further, RANTES has been shown to play an essential role in the recruitment of antigen specific T cells to lung following *M.tb* infection [10] as the absence of RANTES delayed T cell entry into the lung and impaired bacterial control [10]. Based on these data, we opted to evaluate whether cigarette smoke exposure had attenuated the level of RANTES produced in our mycobacterial infection model. To do so, bronchoalveolar lavage fluids (BALF) were collected at the time of sacrifice, and a specific ELISA for RANTES was conducted. In keeping with T cell data, continuous exposure to cigarette smoke significantly attenuated levels of RANTES by >70% (Figure 3G). On the other hand, cigarette smoke cessation partially restored the levels of RANTES although they did not reach the levels seen room air exposed mice (Figure 3G), correlating closely with the relative levels of T cell responses in the lung (Figure 3A–F).

**Figure 4:**
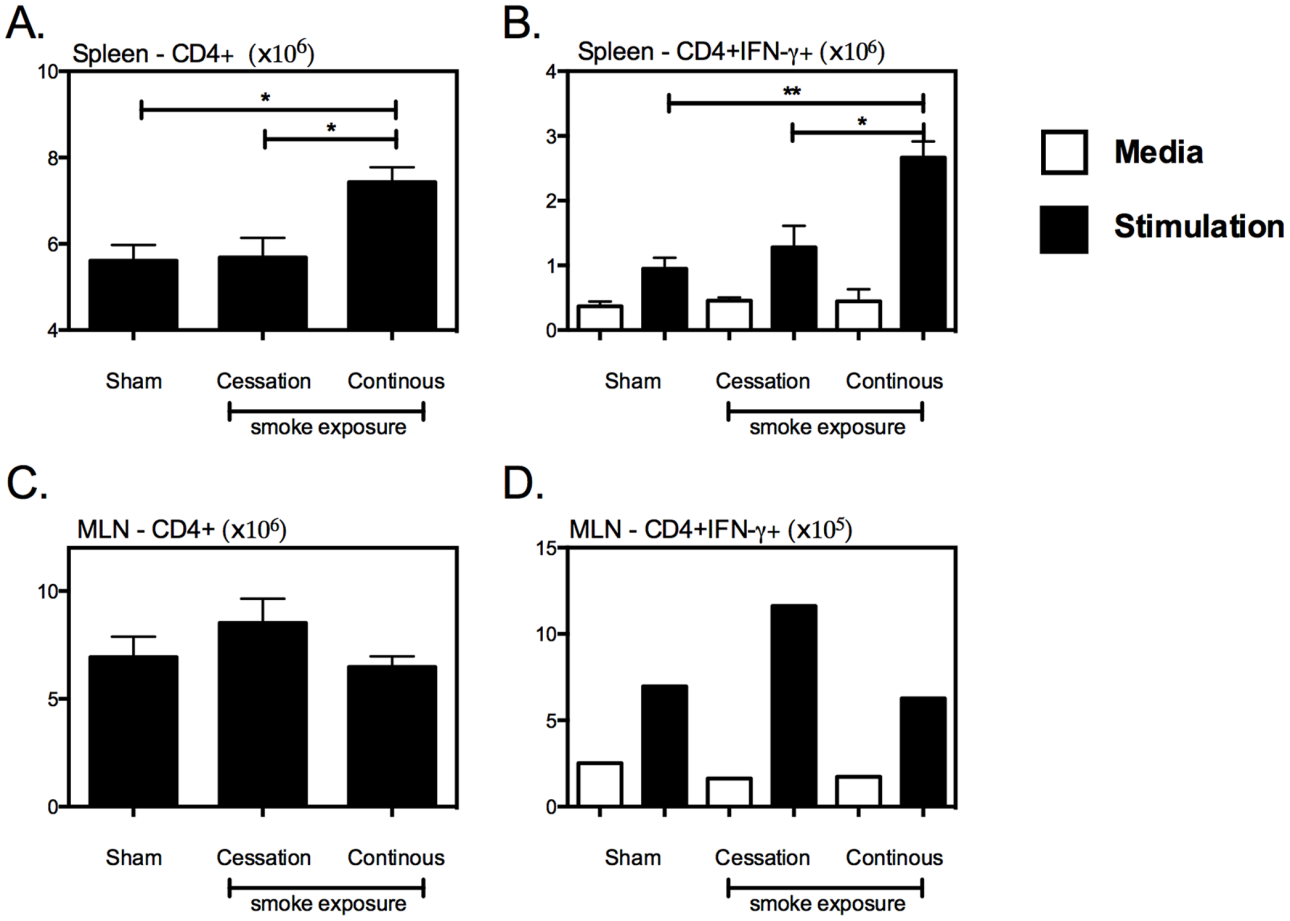
Continuous cigarette smoke exposure does not impair the generation of type 1 immunity in the in the spleen or MLN. Following the exposure-challenge model described in Figure 2A, we evaluated the impact of cigarette smoke exposure on the establishment of type 1 immune response in the spleen and MLN of mycobacterial infected mice. The numbers of CD4+ and CD4+IFN-γ+ T cells were evaluated in the spleen (A&B), and the MLN (C&D). Values represent the mean and standard error for 5 mice per exposure protocol. *p≤0.05; **p≤0.01.

In contrast with the observed T cell deficiency in the lung (Figure 3A–G), continuous cigarette smoke exposure resulted in an increased number of total CD4+ (Figure 4A) and activated CD4+IFN-γ+ T cells (Figure 4B) in the spleen, while having a minimal effect on numbers of T cells in the MLN (Figure 4C/4D), indicating that cigarette smoke exposure impairs the recruitment of CD4+IFN-γ+ to the lung, rather than suppressing their priming in the peripheral lymphoid tissues.

### Continuous Cigarette Smoke Exposure Reduces Type 1 but Enhances Type 2 Cytokine Responses in the Lung

**Figure 5:**
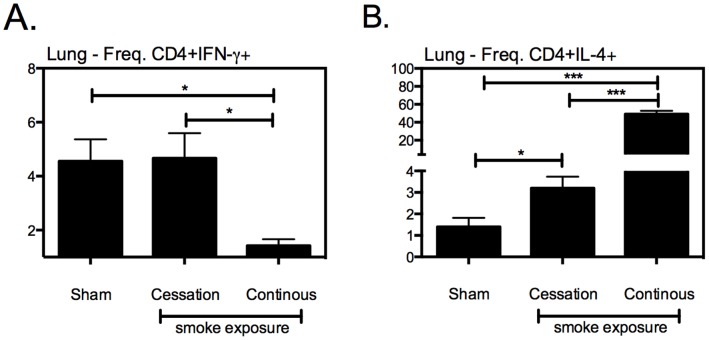
Continuous cigarette smoke exposure alters the balance of Th1 and Th2 CD4 T cells in the lung. Following the exposure-challenge model described in Figure 2A, we evaluated the impact of cigarette smoke exposure on the frequency of CD4+IFN-γ+ (A) and CD4+IL-4+ (B) T cells in the airway lumen of mycobacterial infected mice. Values represent the mean and standard error for 5 mice per exposure protocol. *p≤0.05; **p≤0.01; ***p≤0.001.

Given the remarkable impact of cigarette smoke exposure, particularly the continuous cigarette smoke exposure, on the recruitment of Th1 polarized cells to the lung, we set out to evaluate whether cigarette smoke exposure had altered the balance of Th1 and Th2 T cells in the lung. Of significant interest, while continuous cigarette smoke exposure inhibited the recruitment of CD4+IFN-γ+ T cells to the lung (Figure 5A), it enhanced Th2 CD4+IL-4+ (Figure 5B) responses in the lung, suggesting that cigarette smoke exposure may alter the specific polarization of T cells that enter the lung.

**Figure 6:**
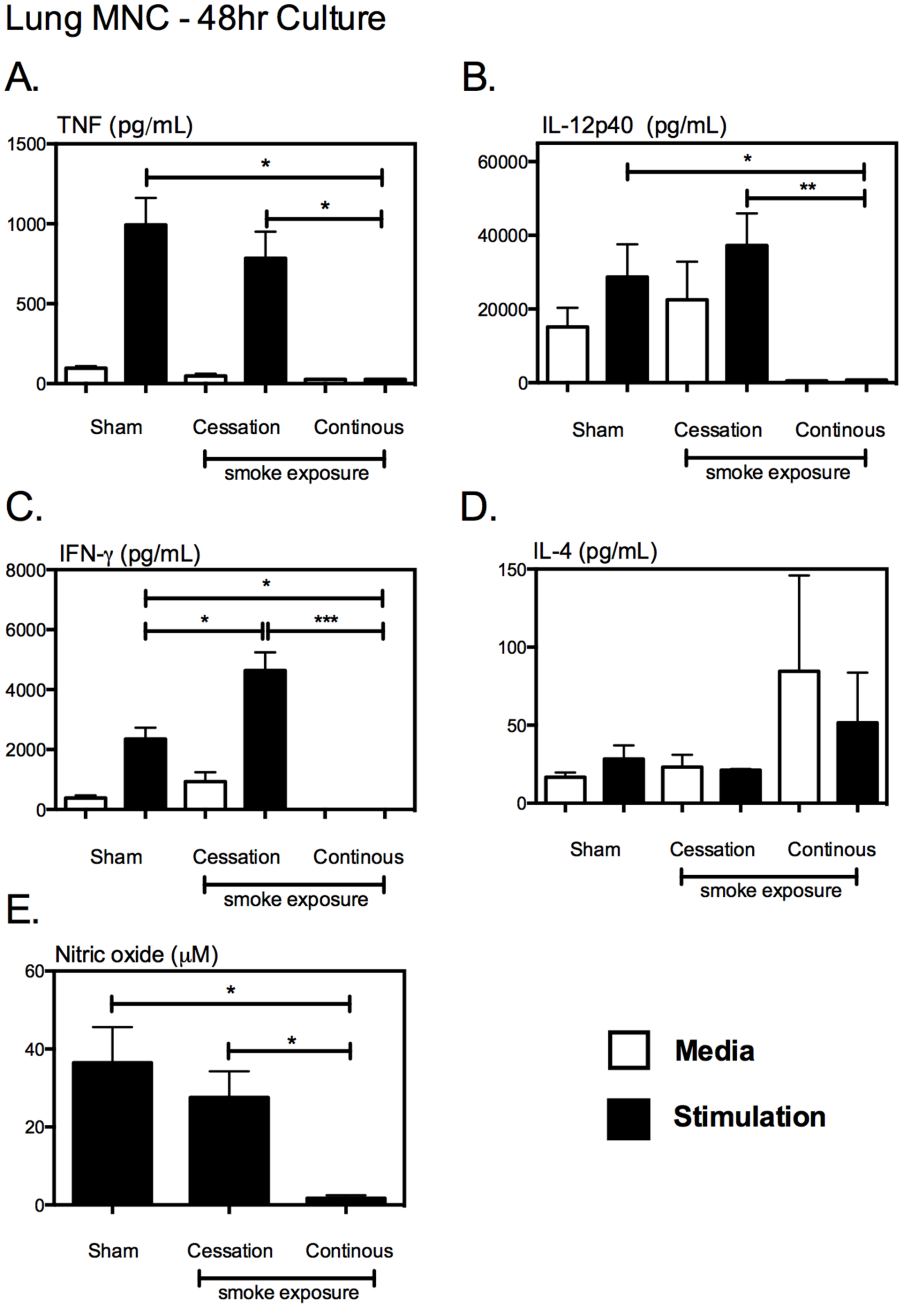
Continuous, but not discontinuous smoke exposure, impairs the production of type 1 cytokines while enhancing the production of IL-4, and reducing the production of bactericidal nitric oxide by lung MNCs following mycobacterial infection. Following the exposure-challenge model described in Figure 2A, we evaluated the impact of cigarette smoke exposure on the production of type 1 &2 cytokines and nitric oxide by mycobacteria infected lung MNCs. Following 48 hr lung MNC culture, the levels of TNF (A), IL-12p40 (B), IFN-γ (C), IL-4 (D) were evaluated by cytokine ELISA, and production of nitric oxide (E) by a modified Griess assay. Values represent the mean and standard error for 5 mice per exposure protocol. *p≤0.05; **p≤0.01; ***p≤0.001.

To further investigate the effect of cigarette smoke exposure on the balance between Th1 and Th2 polarization during mycobacterial infection, lung mononuclear cells (MNC) were isolated from the different exposure groups and subjected to *ex vivo* recall stimulation with crude mycobacterial antigens and following 48 hr culture, supernatants were collected and the production of specific cytokines determine in collected supernatants. Continuous cigarette smoke exposure, but not cigarette smoking cessation, significantly impaired the production of TNF (Figure 6A), and Th1 cytokines IL-12 (Figure 6B), and IFN-γ (Figure 6C), while enhancing the production of Th2 cytokine IL-4 (Figure 6D). Given its critical mycobactericidal activities in infected macrophages [27], we also examined the levels of nitric oxide production. Consistent with severely diminished Th1 cytokine production, continuous cigarette smoke exposure severely hindered the ability of lung MNC to produce nitric oxide (Figure 6E). On the other hand, correlating with relatively unaltered Th1 cytokine responses, cigarette smoking cessation only minimally affected the production of nitric oxide (Figure 6E). Together, these data indicate that continuous cigarette smoke exposure, but not cigarette smoking cessation, markedly dampens the production of Th1 cytokines and bactericidal products in the lung. Thus severely blunted Th1 immunity in the lung by continuous cigarette smoke exposure is the mechanism for weakened mycobacterial control in the lung.

### Continuous Cigarette Smoke Exposure Dampens the Functionality of APC Populations in the Lung, but not in the Spleen or MLN

**Figure 7:**
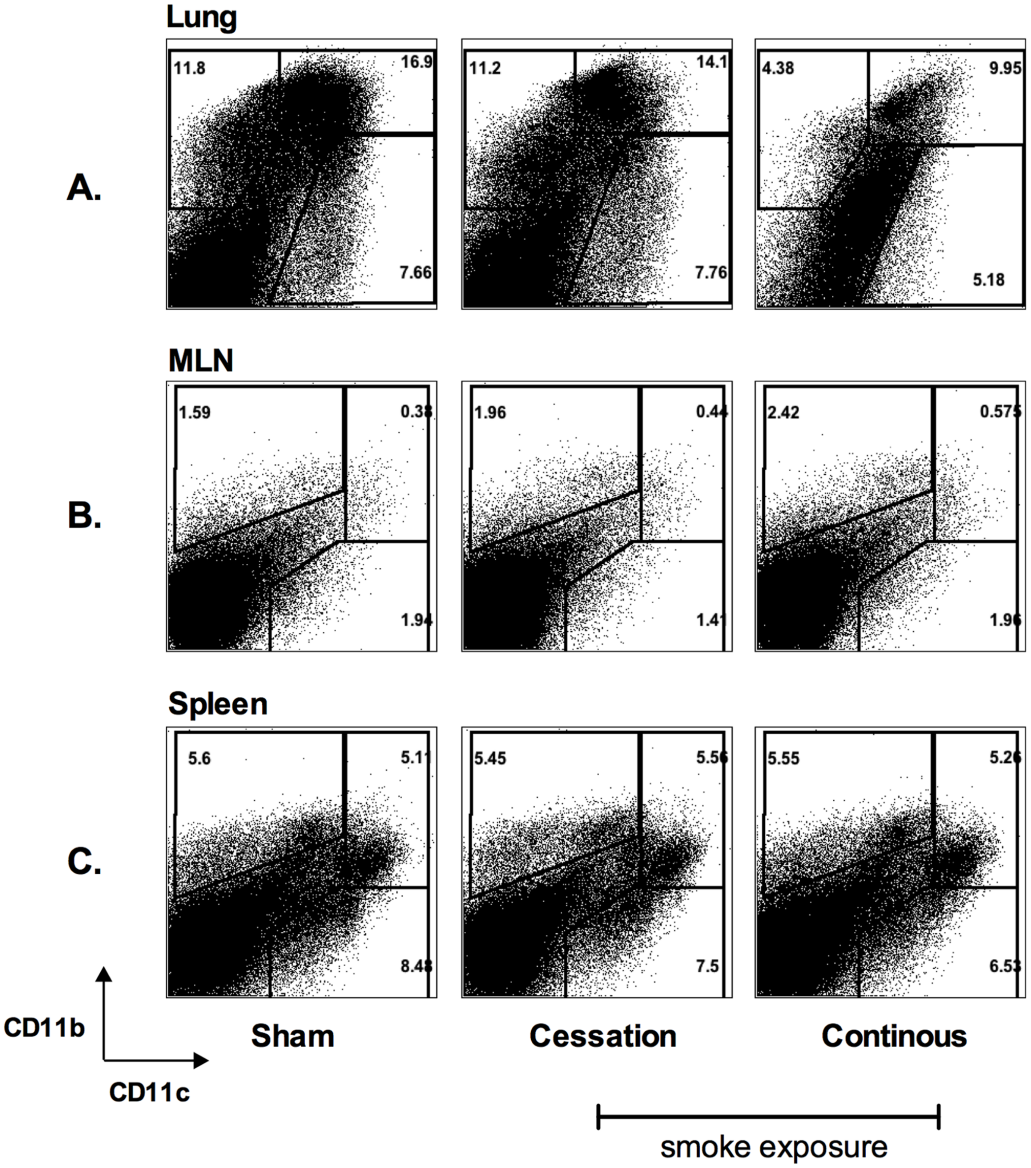
Continuous cigarette smoke exposure alters the surface marker expression of lung, but not spleen or MLN APC populations following mycobacterial infection. Following the exposure-challenge model described in Figure 2A, we evaluated the impact of cigarette smoke exposure on the expression of common APC markers, CD11b+ and CD11c+ in the lung of mycobacterial infected mice. The distribution of CD11b+ and CD11c+ by APC populations of the lung (A), MLN (B) and spleen (C) were evaluated. Panels are representative flow plots for lung mononuclear cells isolated from 5 mice for each exposure protocol.

Thus far we have observed that cigarette smoke exposure, particularly continuous cigarette smoke exposure, suppressed T cell responses in the lung, but had little impact on T cell priming in the mediastinal draining lymph nodes (MLN). In order to understand the potential mechanisms for this divergence in T cell responses, we sought to evaluate the phenotype of various APC populations in the lung, MLN and spleen following mycobacterial infection. Compared to sham room air exposure or cigarette smoking cessation groups, continuous cigarette smoke exposure dramatically reduced the frequency and total numbers of CD11b+CD11c−, CD11b+CD11c+, and CD11b−CD11c+ APCs, indicating a global reduction in the number of APCs in the lungs of these animals following mycobacterial infection (Figure 7A). Of interest, this effect was not seen in the spleen or MLN of continuous cigarette smoke-exposed mice, and the distributions of APC populations in these compartments were similar (Figure 7B/7C). In keeping with the T cell responses, cigarette smoke cessation partially restored the distribution of lung APC populations (Figure 7A), with no notable differences seen in the MLN or spleen (Figure 7B/7C) of the mice of cigarette smoking cessation.

**Figure 8:**
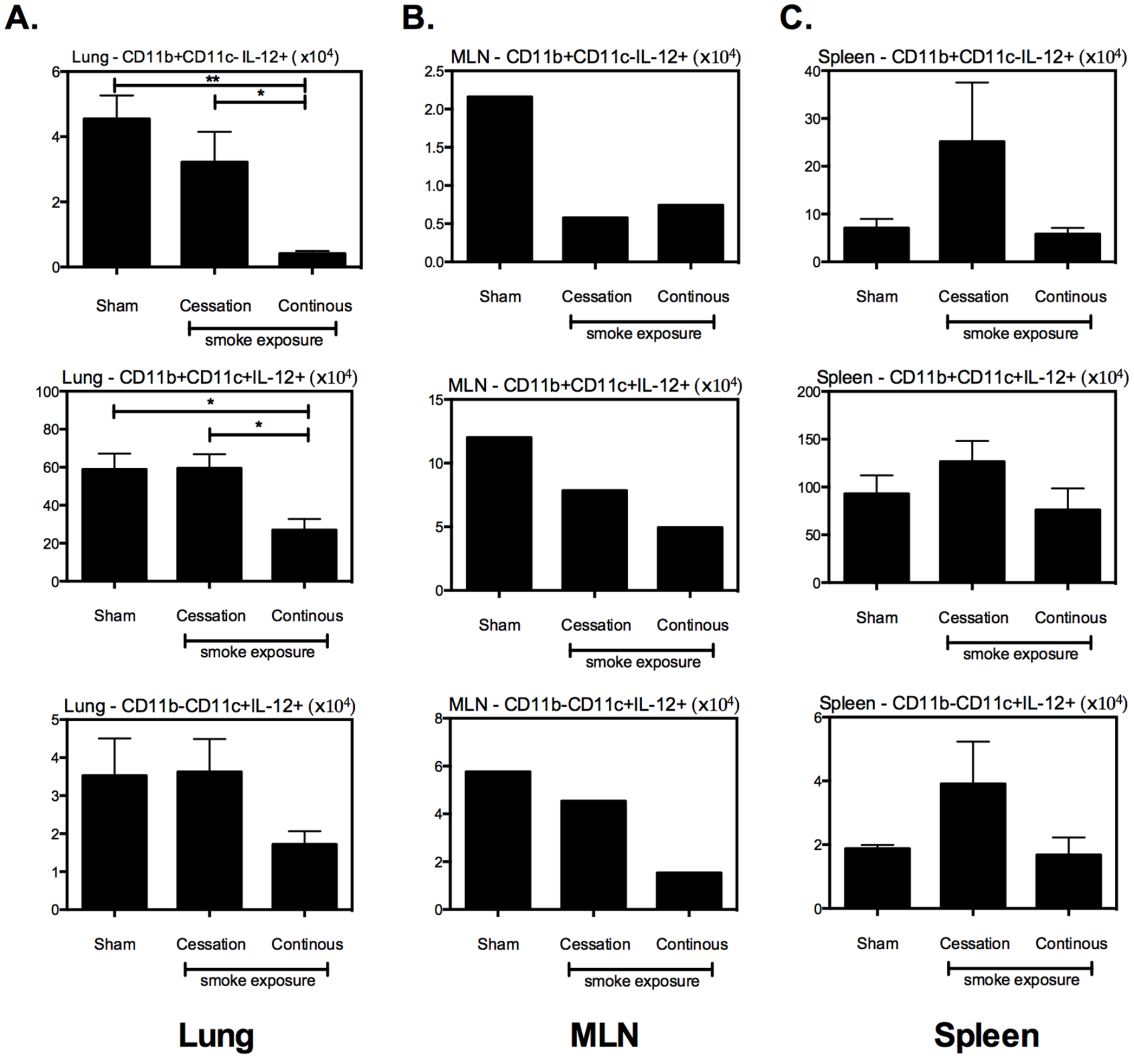
Continuous cigarette smoke exposure suppresses the ability of local, but not systemic, APCs to produce Th1 polarizing cytokines following mycobacterial infection. Following the exposure-challenge model described in Figure 2A, we evaluated the impact of cigarette smoke exposure on the total number of IL-12 & TNF producing APCs in the lung (A), MLN (B), and spleen (C). Values represent the mean and standard error for 5 mice per exposure protocol. *p≤0.05; **p≤0.01.

To determine the functionality of lung APCs, we examined their production of Th1-polarizing cytokines. Following mycobacterial infection, continuous cigarette smoke exposure markedly reduced the numbers of IL-12-producing cells in all lung APC populations analyzed (Figure 8A). Continuous cigarette smoke exposure also similarly reduced IL-12 producers in all APC populations in the MLN (Figure 8B). In comparison, in keeping with T cell responses, cigarette smoking cessation did not reduce IL-12 producers in the lung and only mildly decreased it in the MLN (Figure 8B). Contrast to its profound effect on lung APCs, continuous cigarette smoke exposure had little impact on IL-12-producing APC populations in the spleen while cigarette smoke cessation even somewhat increased such cells in the spleen (Figure 8C). The impact of cigarette smoke exposure on TNF-producing APCs in various tissue compartments was less pronounced (data not shown). Taken together, these data indicate that continuous cigarette smoke exposure, but not cigarette smoking cessation, severely reduces the number and activation of APC populations primarily in the lung with a much less effect in the systemic tissue compartments. These are likely the mechanisms accounting for the blunted T cell responses in the lung and unaltered T cells in the MLN and spleen in the animals that were continuously exposed to cigarette smoke.

## Discussion

Despite strong epidemiological data linking cigarette smoke exposure to the development of active pulmonary TB, the role of cigarette smoke exposure in TB remains to be established. Recent experimental studies have only begun to dissect the relationship of prior cigarette smoke exposure to host anti-mycobacterial immunity [14], [15]. In these studies, the cigarette smoke exposure was discontinued throughout the course of pulmonary mycobacterial infection. Thus to date it has remained completely unknown whether host defense against mycobacterial infection is differentially affected by continuous and discontinuous cigarette smoke exposure, and if so, whether cigarette smoking cessation may help restore the altered host defense.

We set out to address these questions in the models of pulmonary mycobacterial infection established by using both attenuated and virulent strains of mycobacteria. The use of attenuated *M. bovis* BCG strain allowed us to compare the effects of continuous and discontinuous cigarette smoke exposure on anti-mycobacterial immunity. Specifically, using this model, after mycobacterial challenge the prior cigarette smoke-exposed animals were continuously exposed to cigarette smoke on a daily basis. Continuous cigarette smoke exposure post-*M.tb* infection is unfeasible within the P3 biohazard confinement facility and is made possible only when an attenuated mycobacterial species is used for challenge. However, we used a virulent strain of *M.tb* (H_37_Rv) to verify the protection result that compared to continuous exposure, a 4-wk cigarette smoking cessation improved immune protection from pulmonary TB. Of importance, we extended this observation and found that a 6-wk cigarette smoking cessation completely restored anti-TB immune protection to the level seen in sham room air-exposed animals. Despite the virulent nature of *M.tb* H_37_Rv, there is significant variability in the virulence and inflammatory responses mounted by various region-specific clinical strains. Future studies may examine the potentially differential impact of cigarette smoke on the outcome associated with these strains [28]. Regardless, our findings hold significant implications to anti-cigarette smoking campaign, suggesting that smoking cessation is beneficial to restoring lung host defense mechanisms against pulmonary TB.

While continuous cigarette smoke exposure profoundly impacts local immunity within the lung, we did not note any alteration to the generation of CD4+IFN-γ+ T cells in the peripheral lymphoid organs of mycobacterial*-infected* mice. We demonstrate that whereas continuous cigarette smoke exposure profoundly alters the local lung immune environment attenuating the release of critical anti-mycobacterial cytokines, IL-12, TNF and IFN-γ and the T cell chemokine RANTES, cigarette smoking cessation moderated these affects. Given that a loss of any one of these cytokines can severely compromised mycobacterial immunity, it is interesting that the phenotype seen following cigarette smoke exposure is unique to any one deficiency model. To draw comparison, in the absence of IL-12, mycobacterial infected mice fail to mount Th1 responses, fail to control bacterial growth, and fail to localize T cells to lung, an observation shared with cigarette smoke exposure [9]. Similarly, TNF and IFN-γ are synergistically required for the production of nitric oxide without which bacterial dissemination rapidly occurs. Moreover, the absence of RANTES severely attenuates the recruitment of T cells to lung, impairing bacterial control [10]. Despite their similarities it should be noted that the development of lung pathology significantly differs between these deficiencies, with IL-12 and RANTES deficient mice displaying less lung pathology, while TNF and IFN-γ deficient mice displaying exaggerated lung pathology. The difference lies in the recruitment of T cells to the lung, where IL-12 and RANTES deficient mice recruit far fewer active T cells, TNF and IFN-γ deficient mice recruit far more, likely in an attempt to compensate for the impaired ability of the infected APCs to produce nitric oxide. While cigarette smoke impairs bacterial control we attribute the decreased lung pathology to the reduced recruitment of T cells to lung, similar to what has been documented with IL-12 and RANTES deficiencies. It is interesting that cigarette smoke so profoundly influences multiple anti-mycobacterial immune pathways, reducing APC’s ability to recruit and maintain protective CD4+IFN-γ+ T cells in the lung, while simultaneously attenuating pathology. Furthermore, it should be noted that while cigarette smoke exposure significantly blocked T cell recruitment to lung, those T cells that did get recruited displayed enhanced Th2 responses, promoting an increased frequency of Th2 CD4+ IL-4+ T cells, and higher levels of IL-4. Not only did continuous cigarette smoking attenuate the establishment of Th1 immunity but it also augmented Th2 immunity, thus likely further impairing the host’s ability to control mycobacteria. While the reduced production of RANTES likely contributed to the defective accumulation of T cells in the lung, it remains plausible that the unique environment created by cigarette smoke may have negatively impacted the survival of recruited T cells causing them to undergo apoptosis or necrosis. Moreover, while not addressed in this study cigarette smoke may have generated a suppressive population of T regulatory cells capable of attenuating Th1 immunity in the lung. Taken together, the unique influence of cigarette exposure on the development of mycobacterial immunity cannot be attributed to its effect on a single component of the host immune response, but rather it is due to its broad impact on the innate, and ensuing adaptive immune cells locally residing in the lung.

Our study for the first time demonstrates that sufficient cigarette smoke cessation restores protective immunity to *M.tb* challenge by reestablishing APC functionality, and promoting the recruitment of CD4+IFN-γ+ T cells to the lung. Conceivably, the enhanced recruitment of CD4+IFN-γ+ T cells can be attributed to increased levels of RANTES produced following smoking cessation. Moreover, the increased presence of IFN-γ-producing T cells likely contributed to the production of nitric oxide and enhanced bacterial control. These observations provide an explanation for the rapid recovery and restoration of TB immunity seen clinically in humans following cigarette smoking cessation [29]. Improved TB protective immunity in the lung by cigarette smoking cessation was further demonstrated in our BCG immunization model. Of note, such improvement appears even more robust in BCG-immunized animals than in unimmunized counterparts as the *M.tb-*challenged BCG-vaccinated mice of 4 wk smoking cessation had similarly improved protection as those of sham room air exposure. Although it is unfeasible to carry out such a study, conceivably continuous cigarette smoke exposure in *M.tb*-challenged BCG-immunized animals would have produced a different outcome, as has been documented clinically [4], [5], [6]. Nonetheless, the observation that cigarette smoking cessation allows the BCG-immunized hosts to even more quickly restore lung protection is highly relevant to TB endemic areas where BCG vaccination is routinely carried out in childhood. These findings together further support the view that cigarette smoking cessation will help control the global TB epidemic.

Our study further reveals that continuous cigarette smoke exposure results in much less lung granulomatous inflammation, in keeping with impaired innate and adaptive immune responses in the lung. This observation is highly significant as the majority of TB symptoms are due to the inflammatory responses generated by the host. Particularly alarming is the notion that cigarette smoke mediated - inflammatory suppression may allow for the infected host to remain asymptomatic despite active bacterial growth. Indeed, epidemiological data suggests that smokers are 9 times more likely to die of active TB than non-smokers, with the vast majority (83%) having no TB-like symptoms prior to the onset of disease [29]. This sharply contrasts non-smokers where mild TB-like symptoms are generally reported significantly before the onset of disease [29]. The notion that cigarette smoking may mask TB symptoms, allowing a critical bacterial threshold to be reached before diagnosis, may explain why the likelihood of mortality is so much higher in TB-infected smokers. Together, our findings imply that cigarette smoke exposure has the capacity to augment the lethality of this deadly pathogen by impairing host mechanisms of bacterial control.

In summary, our data demonstrates that cigarette smoke impacts anti-TB immunity largely through impairing the recruitment and maintenance of Th1 T cells in the lung, rather than impairing systemic T cell priming. Furthermore we have shown that cigarette smoke exposure must be maintained for its immunosuppressive effects to persist, where cigarette smoke cessation restores chemotactic signals, promoting the recruitment of T cells to lung, vastly improving bacterial control. Therefore, we have experimentally provided novel information on how cigarette smoke exposure impacts the establishment of anti-mycobacterial immunity in the lung, and why protective immunity to *M.tb* can be rapidly restored following cigarette smoking cessation. Our findings suggest that one of the effective ways to avoid/combat active TB is to stop cigarette smoking. Such recommendation is further supported by clinical observation that continued cigarette smoke exposure not only suppresses host immunity but also hinders the effect of anti-TB antibiotic therapy [30].

## Supporting Information

Figure S1 **Cigarette smoke exposure recruits various immune cell populations and causes pronounced alterations to the lung structure.** Following 6 wks of cigarette smoke (or room air) exposure mice were sacrificed and their lungs removed and bronchoalveolar lavage performed (A). One lobe of the collected lung was used for mononuclear cell isolation, and the remaining were sectioned and stained with H&E for the assessment of gross pathology. Cigarette smoke exposure altered the percentage (B) and absolute numbers (C) of various immune cells infiltrating the airway lumen. Lung histological sections revealed pronounced structural changes were induced by cigarette smoke (cs) exposure (D). Specifically, cs resulted in increased alveolar space (E), inflammation of the alveolar septum (F), and moderate epithelial damage (G). Differential cell counts represent the mean frequencies and total numbers of 5 room air and 5 cigarette smoke exposed mice. Specific sections displayed for the assessment of gross pathology are representative of each exposure group.
(TIFF)

Figure S2 **Cigarette smoke exposure alters surface marker expression on lung APC populations.** Following 6 wks of cigarette smoke (or room air) exposure mice were sacrificed and their lungs removed and processed for mononuclear cell isolation. Specific changes in the expression of APC markers were determined by flow cytometry. Representative flow plots for lung mononuclear cells isolated from 5 individual room air (A) or cs exposed mice (B). Samples were stained for CD11b, CD11c, GR1 and F4/80 to determine the change in specific lung APC populations.
(TIFF)

Figure S3 **Prior** **cigarette smoke exposure does not impair BCG vaccine efficacy following** ***M.tb*** **challenge.** Following subcutaneous BCG immunization, mice were exposed for a period of 6 wks to cigarette smoke (or room air). Following cigarette smoke exposure immunized and unimmunized mice were subjected to *M.tb* H_37_Rv challenge. At the time of challenge cigarette smoke exposure was discontinued (A). *M.tb* infected, prior cigarette smoke exposed BCG vaccinated mice were compared to room air- unimmunized and immunization controls. The bacterial burden following the various exposure protocols was determined by colony formation assay in the lung and spleen of infected mice (B&C), and the histological impact on lung pathology determined by H&E staining of lung sections (D–F). CFU numbers represent the mean and standard error of 5 mice exposed to either, continuous cigarette smoke, or room air and BCG immunized. Selected histological sections are representative of the independent groups with 5 mice per exposure protocol. Values *p≤0.05; **p≤0.01.
(TIFF)
