# Supplementary figures and images for: Continuous and Discontinuous Cigarette Smoke Exposure Differentially Affects Protective Th1 Immunity against Pulmonary Tuberculosis
Source: PLoS One. 2013 Mar 19;8(3):e59185. doi: 10.1371/journal.pone.0059185 (PMC3602464; doi:10.1371/journal.pone.0059185)

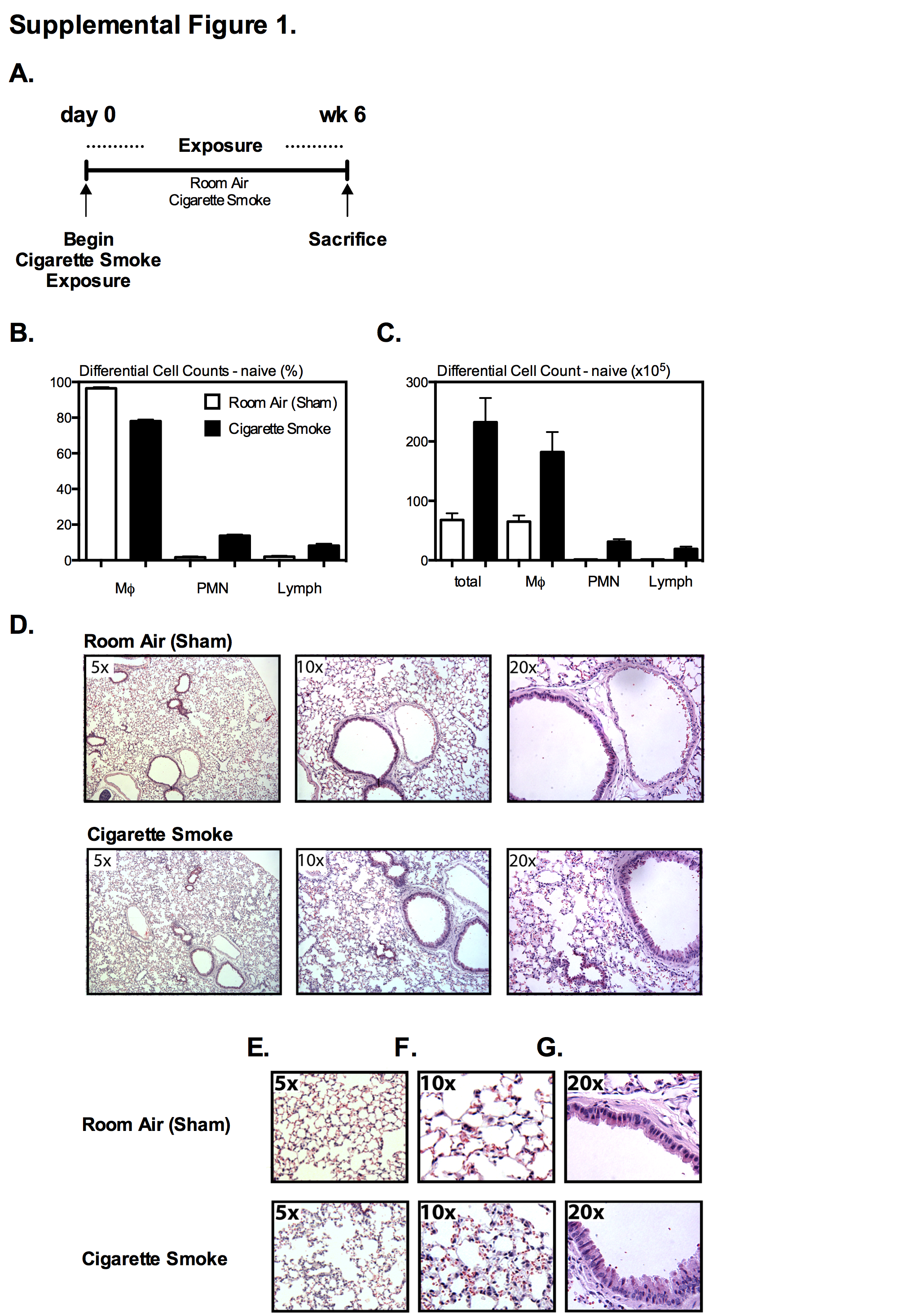

Supplement: Figure S1 — Cigarette smoke exposure recruits various immune cell populations and causes pronounced alterations to the lung structure. Following 6 wks of cigarette smoke (or room air) exposure mice were sacrificed and their lungs removed and bronchoalveolar lavage performed (A). One lobe of the collected lung was used for mononuclear cell isolation, and the remaining were sectioned and stained with H&E for the assessment of gross pathology. Cigarette smoke exposure altered the percentage (B) and absolute numbers (C) of various immune cells infiltrating the airway lumen. Lung histological sections revealed pronounced structural changes were induced by cigarette smoke (cs) exposure (D). Specifically, cs resulted in increased alveolar space (E), inflammation of the alveolar septum (F), and moderate epithelial damage (G). Differential cell counts represent the mean frequencies and total numbers of 5 room air and 5 cigarette smoke exposed mice. Specific sections displayed for the assessment of gross pathology are representative of each exposure group. (TIFF) [file pone.0059185.s001.tiff]

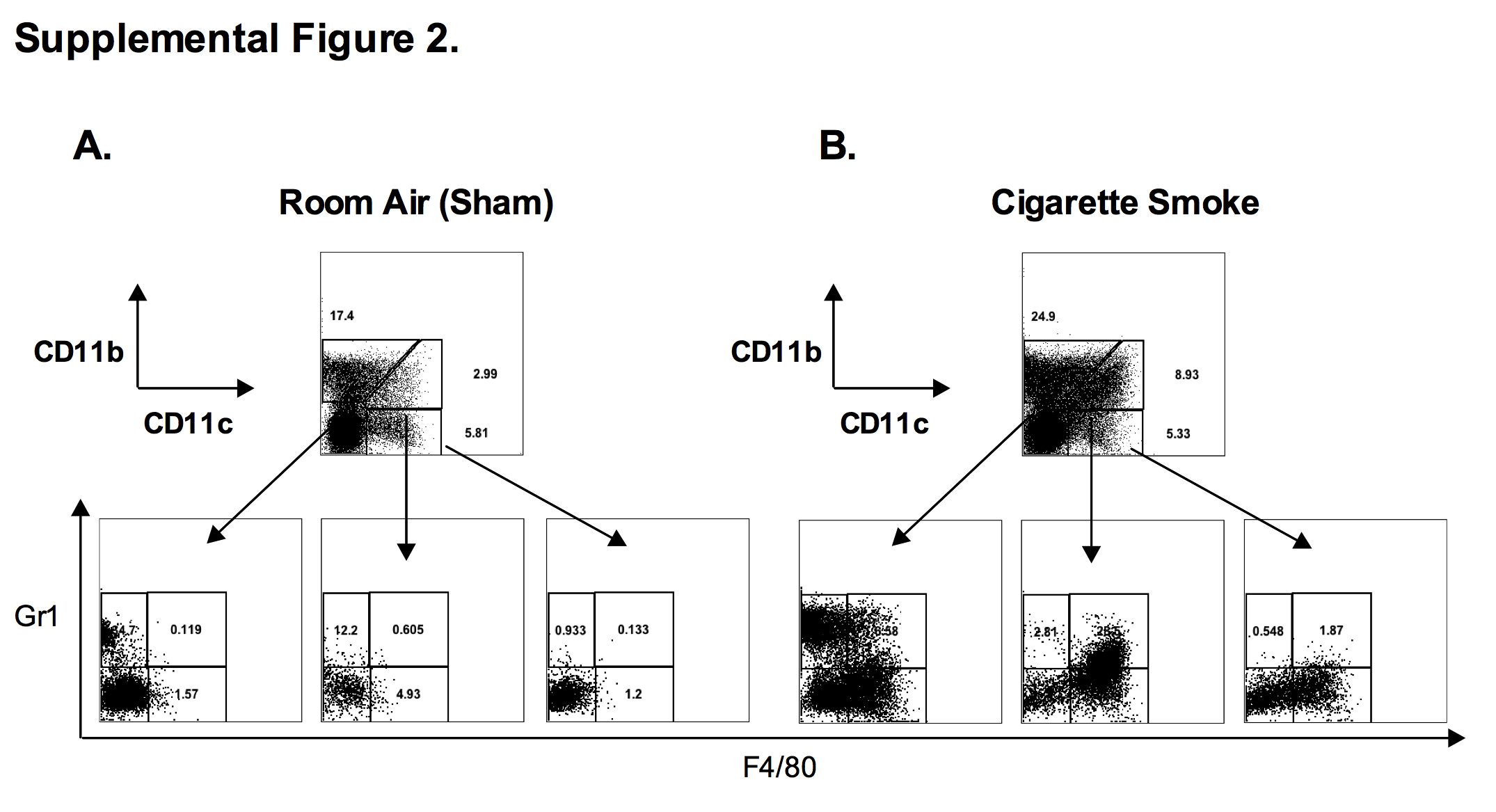

Supplement: Figure S2 — Cigarette smoke exposure alters surface marker expression on lung APC populations. Following 6 wks of cigarette smoke (or room air) exposure mice were sacrificed and their lungs removed and processed for mononuclear cell isolation. Specific changes in the expression of APC markers were determined by flow cytometry. Representative flow plots for lung mononuclear cells isolated from 5 individual room air (A) or cs exposed mice (B). Samples were stained for CD11b, CD11c, GR1 and F4/80 to determine the change in specific lung APC populations. (TIFF) [file pone.0059185.s002.tiff]

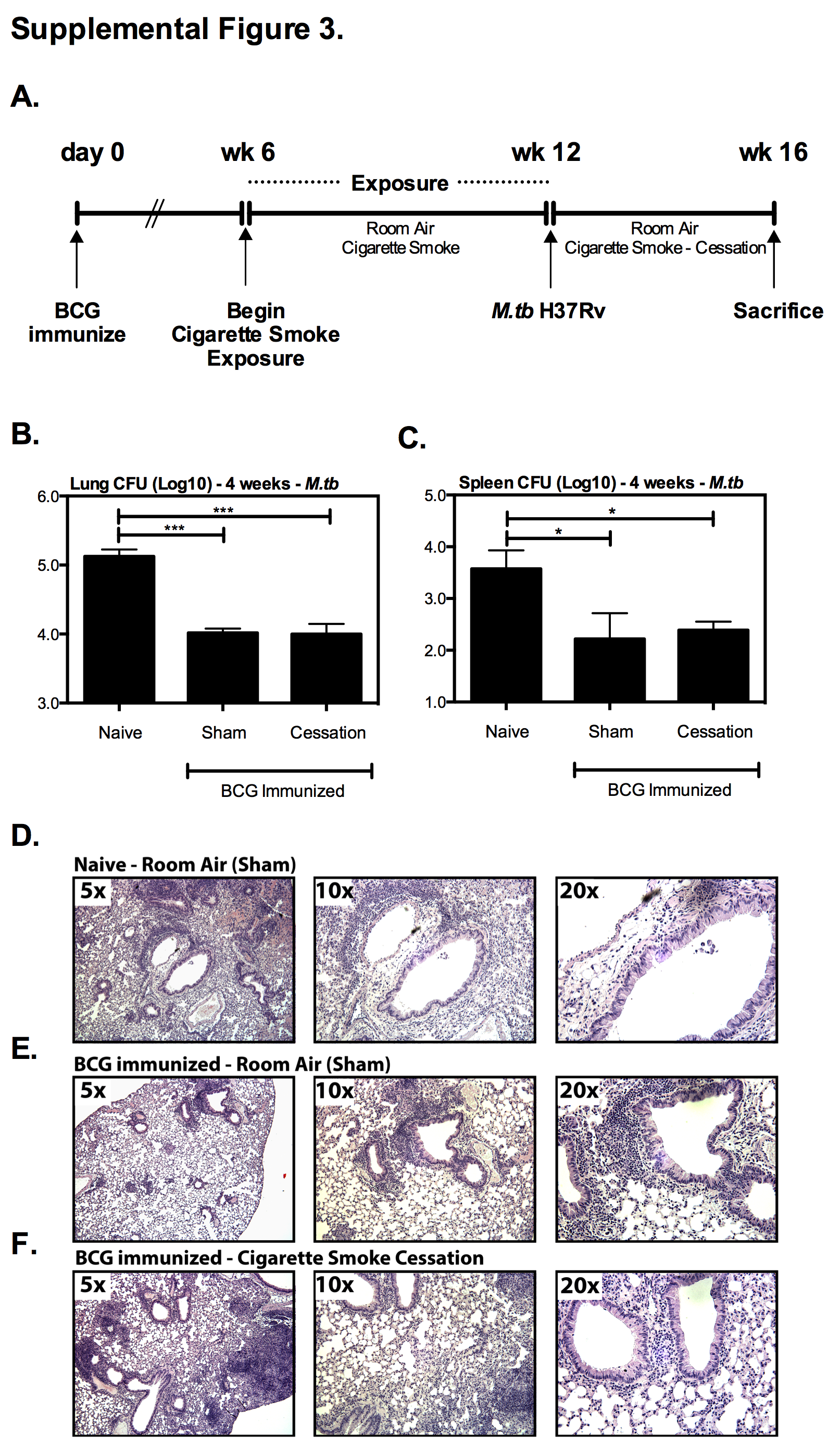

Supplement: Figure S3 — Prior cigarette smoke exposure does not impair BCG vaccine efficacy following M.tb challenge. Following subcutaneous BCG immunization, mice were exposed for a period of 6 wks to cigarette smoke (or room air). Following cigarette smoke exposure immunized and unimmunized mice were subjected to M.tb H37Rv challenge. At the time of challenge cigarette smoke exposure was discontinued (A). M.tb infected, prior cigarette smoke exposed BCG vaccinated mice were compared to room air- unimmunized and immunization controls. The bacterial burden following the various exposure protocols was determined by colony formation assay in the lung and spleen of infected mice (B&C), and the histological impact on lung pathology determined by H&E staining of lung sections (D–F). CFU numbers represent the mean and standard error of 5 mice exposed to either, continuous cigarette smoke, or room air and BCG immunized. Selected histological sections are representative of the independent groups with 5 mice per exposure protocol. Values *p≤0.05; **p≤0.01. (TIFF) [file pone.0059185.s003.tiff]
